# The State Registration of Nurses

**Published:** 1913-05-03

**Authors:** Sydney Holland

**Affiliations:** Chairman, London Hospital, Whitechapel, E.


### The Editor's Letter-Box.

### The State Registration of Nurses.

*To the Editor of* The Hospital.

Dear Sir,—I do not ask for space to deal with the question of the State Registration of Nurses. If anyone cares to know the arguments against this mischievous proposal I shall be glad to send him or her a short statement containing those arguments. They will take ten minutes to read and a lifetime to forget.

But I do ask space to contradict the statement made by Dr. Chapple that nurses are ever sent from the London Hospital before they have been through the full training. Full training is obtained at the London Hospital in two years. He said that he knew of a nurse who was sent out after only one year and ten months' training, and I have wired to him, paid reply, and hunted him all over London to get particulars of this case—all in vain. It is simply untrue.

Abuse of the London Hospital is one of the weapons of the Registration leaders. They find it is difficult to account for the universally recognised merits of its nurses. Well may Mr. Asquith have exclaimed, "Do you mean to say we do not get good nurses from the London Hospital?" He knew better, and so do other members of his Cabinet.

How utterly foolish it would be of the London Hospital to change its period of training to three years when twenty-three years' experience has shown that we can train a nurse perfectly in two years! And it is suggested that we should make this change at the cry of those who, for want of method, opportunity, or organisation, say they cannot train a nurse in three years! I happen to be chairman of two other hospitals. At one it certainly takes three years to train a nurse, at the other we could not train a nurse in thirty years.

It is not "time" any more than "passing an examination" which makes a woman into a nurse, but opportunity for gaining practical knowledge, careful individual teaching, and an organisation which sees that during her training she has a sufficiently varied experience.

At the London Hospital we have, to help us in our training, the finest preliminary nurse-training school in the world. Women enter our wards to learn nursing after having passed through this course of preliminary training. They have learned much during the time spent at Tredegar House, which it would take far longer to learn, less well, in the wards of a hospital.

I remember Miss Florence Nightingale saying to me that if she could give her whole time to a good woman and had suitable opportunities she could make her into a competent nurse in six months.

It is too late now to question the reputation of London Hospital nurses, or the efficiency of their training. Nurses with our training have been selected to nurse his late Majesty and many members of the Royal Family. They are holding some of the most responsible posts all over the world. Twenty-one are actually matrons of hospitals in London alone. Several of our nurses with only this "miserably insufficient" training earned the Royal Red Cross in South Africa, and to-day the Matron-in-Chief of the Army and both principal matrons hold the London Hospital Certificate. The Local Government Board have just appointed a second "Londoner" as another of their

Inspectors, not to speak of the "Londoners" who are filling many other important appointments.

The 253 nurses on our private staff are in constant employment, and eagerly sought after by the best doctors in the land, and by the nicest people. Already this year we have been compelled to refuse 585 cases.

All this is just what the advocates of State registration cannot get over, and so they have recourse to inaccurate statements like those of Dr. Chapple with regard to the pay of our private nurses and to my motives in opposing State registration.

Sir Victor Horsley said that I had spoken at a meeting of nurses and found it hostile. Does that prove I am wrong? If so, Sir Victor Horsley must seldom be right in his campaigns. But, as a fact, on the occasion he refers to no vote was taken. He also said that the names of those who opposed State registration were kept secret. I cannot imagine what he means. Has he never seen the protest signed by 277 matrons, 91 chairmen and other hospital workers, by 1,300 nurses and 300 medical men—a list we could add to enormously if it were worth while? He is welcome to a copy of the protest. He complains that we will not meet him in the open. There is some truth in that, but not as regards myself. Women engaged daily in hospital work, even if they had the time, are seldom qualified or able to speak at meetings, or care to do so. Some may prefer to be quiet rather than face the treatment by one section of the nursing press, which the Matron of St. Bartholomew's has lately had to put up with because she dared to be against State registration. We do not happen to have on our side a lot of ex-matrons or an editor of a nursing journal with time at her disposal.

Mr. Asquith suggested a compromise. One is possible. An official directory, which would give to everyone the certain knowledge what training any woman had had who called herself a nurse and whom he thought of employing. This official directory would give to neither side what they want. But it could be passed unopposed if it is thought that the public needs more protection from unqualified nurses than it could obtain from the inquiry which ought to be made before any nurse is engaged.—Faithfully yours,

Sydney Holland,
Chairman, London Hospital.

London Hospital, Whitechapel, E.,
April 20, 1913.

